# A new introduced species in South America: the Asian water bug *Synaptonecta issa* (Distant, 1910) (Heteroptera, Micronectidae)

**DOI:** 10.3897/BDJ.14.e186680

**Published:** 2026-07-20

**Authors:** Jonathan David Sanchez-Muelas, Ranulfo González Obando, Karol Viviana Salazar-Rivera, Julianna Freires Barbosa, Felipe Ferraz Figueiredo Moreira

**Affiliations:** 1 Laboratório de Entomologia, Fundação Oswaldo Cruz, Rio de Janeiro, Brazil Laboratório de Entomologia, Fundação Oswaldo Cruz Rio de Janeiro Brazil; 2 Grupo de Investigaciones Entomológicas, Universidad del Valle, Cali, Colombia Grupo de Investigaciones Entomológicas, Universidad del Valle Cali Colombia; 3 Laboratório de Entomologia, Universidade Federal do Rio de Janeiro, Rio de Janeiro, Brazil Laboratório de Entomologia, Universidade Federal do Rio de Janeiro Rio de Janeiro Brazil

**Keywords:** aquatic insects, Colombia, Corixoidea, Pacific Region, truly aquatic bugs

## Abstract

**Background:**

Corixoidea is the only group of water bugs (Hemiptera, Heteroptera, Nepomorpha) that does not exhibit strictly predatory habits. Two families have been recorded from the Neotropics: Corixidae and Micronectidae. The latter is represented by three genera in the region: *Monogobia* Nieser & Chen, 2006, *Synaptogobia* Nieser & Chen, 2006 and *Tenagobia* Bergroth, 1899. At least four species of Corixoidea have been introduced into non-native areas: *Micronecta ludibunda* Breddin, 1905 to North America; *Synaptonecta issa* (Distant, 1910) to North America, the Caribbean Islands and New Zealand; *Trichocorixa verticalis* (Fieber, 1851) to Europe, Africa and New Caledonia; and *T. reticulata* (Guerin-Meneville, 1857) to Hawaii.

**New information:**

Here, we record *S. issa* from South America for the first time. We collected specimens in the Municipality of Buenaventura, Department of Valle del Cauca, Pacific Region of Colombia. We found it in a permanent freshwater lagoon, in areas dominated by the aquatic plant *Cabomba cf. haynessi*. We also found the native Neotropical micronectid *Tenagobia signata* (White, 1879) at the same site and report its first record from Colombia. The introduction pathway of *S. issa* into South America and any environmental impacts that it might cause locally remain unknown and should be investigated.

## Introduction

Water-boatmen (Corixoidea) constitute the only group of truly aquatic bugs (Hemiptera, Heteroptera, Nepomorpha) that are not strictly predatory, given that they can also feed on small organic particles (Barbosa and Rodrigues 2015). However, the diets of taxa such as Heterocorixinae (Corixidae) and Micronectidae remain unknown (Nieser and Chen 2006). Corixoidea is currently comprised of the families Diaprepocoridae, Corixidae and Micronectidae (Zhen et al. 2023), of which the last two have representatives in the Neotropics (Moreira et al. 2018). Micronectidae is divided into the following two subfamilies: the worldwide distributed Micronectinae, with six genera and the Neotropical endemic monogeneric Synaptogobiinae. Two genera of Micronectinae are native to the Neotropical Region, namely *Monogobia* Nieser & Chen, 2006 and *Tenagobia* Bergroth, 1899 (Nieser and Chen 2006).

At least four species of Corixoidea have been introduced to non-native areas. The most widespread and studied species is *Trichocorixa verticalis* (Fieber, 1851) (Corixidae, Corixinae), originally distributed in North America and the Caribbean and subsequently recorded from New Caledonia (Jansson 1982), South Africa (Jansson and Reavell 1999), Portugal (Sala and Boix 2005), Morocco (Taybi et al. 2020) and Spain (Millán et al. 2021). It has the potential to spread into coastal areas of the Mediterranean Sea, Australia and Southeast Asia, as well as Argentina and Uruguay in South America (Guareschi et al. 2013). *Trichocorixa reticulata* (Guerin-Meneville, 1857), a corixid from the Americas, is also recorded as a non-native species in Hawaiian estuaries (Brock and Bailey‐Brock 2007, Carlton and Eldredge 2009). The Micronectidae exotic species are the Oriental *Micronecta ludibunda* Breddin, 1905 (also Oceanian) and *Synaptonecta issa* (Distant, 1910) (Micronectidae, Micronectinae). Both have been recorded from Florida (Polhemus and Rutter 1997, Polhemus and Golia 2006) and the latter also from New Zealand (Jansson and Meyer-Rochow 1990) and the Dominican Republic (Payano-Mercado et al. 2021). In this study, we report *S. issa* in South America for the first time, based on specimens collected in the Chocó Biogeographic Region (Pacific Rainforest). We also report the native Neotropical *Tenagobia signata* (White, 1879) for the first time in Colombia and provide an updated key to Micronectidae genera recorded from region.

## Materials and methods

We used D-shaped aquatic nets to collect, actively moving the net through the aquatic vegetation in the accessible lakes near Dagua River, in Buenaventura Municipality, Pacific Region of Valle del Cauca Department, Colombia. All Micronectidae obtained were preserved in 96% ethanol and brought to the laboratory for identification.

We identified specimens at genus level using the keys provided by Nieser and Chen (2006) and Moreira et al. (2018). Then, we identified species using keys, descriptions and figures of *Synaptonecta* provided by *Lundblad (1933)*, Hutchinson (1940) and Ha and Tran (2021) and of *Tenagobia* provided by *Deay (1935)* and Nieser (1977). To examine the male genitalia, we dissected and mounted them on permanent slides with Canada balsam. We used a Canon T5i camera attached to a Nikon H550S optical microscope and the Helicon Focus 6 software to obtain photographs of specimens and genitalia. We used QGIS 3.32.2 to produce the maps. Specimens collected were deposited in Museo de Entomología de la Universidad del Valle (MUSENUV).

## Taxon treatments

### Synaptonecta issa

(Distant, 1910)

34B348D8-3349-597B-A33D-01436BC5A5FC

#### Materials

- **Type status:** Other material. **Occurrence:** catalogNumber: MUSENUV33939; recordedBy: J.D. Sanchez-Muelas | K.V. Salazar-Rivera | D. Hernández-Romero | R. González; individualCount: 6; sex: 2 males, 1 female; lifeStage: 3 adults, 3 juveniles; occurrenceStatus: present; occurrenceID: CB4927DB-2B22-5741-AD63-D3A6CA613B1D; **Taxon:** scientificName: Synaptonecta issa; kingdom: Animalia; phylum: Arthropoda; class: Insecta; order: Hemiptera; family: Micronectidae; genus: Synaptonecta; taxonRank: issa; taxonomicStatus: Species; **Location:** continent: South America; country: Colombia; countryCode: CO; stateProvince: Valle del Cauca; county: Buenaventura; locality: Citronela; verbatimLocality: Humedal. Punto 1; verbatimElevation: 27; locationRemarks: wetland near the road to Dagua River; verbatimCoordinates: 3°51'52"N 76°58'02"W; decimalLatitude: 3.86455; decimalLongitude: -76.967145; geodeticDatum: WGS84; georeferenceProtocol: GPS; **Identification:** identifiedBy: J.D. Sanchez-Muelas | J.F. Barbosa; dateIdentified: 2024; **Event:** samplingProtocol: Aquatic D Shaped Net; eventDate: 14/10/2023; year: 2023; month: 10; day: 14; **Record Level:** language: en; institutionID: 890.399.010-6; collectionID: RNC:77; institutionCode: Museo de entomología de la Universidad del Valle (MUSENUV); collectionCode: MUSENUV; basisOfRecord: PreservedSpecimen; **Material Entity:** preparations: whole animal (EtOH)
- **Type status:** Other material. **Occurrence:** catalogNumber: MUSENUV33940; recordedBy: J.D. Sanchez-Muelas | J. Hernández-Benavides | R. González; individualCount: 2; sex: 1 male, 1 female; lifeStage: adult; occurrenceStatus: present; occurrenceID: C5676151-9FA5-5DD6-91FA-01F7FA14FE73; **Taxon:** scientificName: Synaptonecta issa; kingdom: Animalia; phylum: Arthropoda; class: Insecta; order: Hemiptera; family: Micronectidae; genus: Synaptonecta; taxonRank: issa; taxonomicStatus: Species; **Location:** continent: South America; country: Colombia; countryCode: CO; stateProvince: Valle del Cauca; county: Buenaventura; locality: Citronela; verbatimLocality: Humedal. Punto 1; verbatimElevation: 27; locationRemarks: wetland near the road to Dagua River; verbatimCoordinates: 3°51'52"N 76°58'02"W; decimalLatitude: 3.86455; decimalLongitude: -76.96715; geodeticDatum: WGS84; georeferenceProtocol: GPS; **Identification:** identifiedBy: J.D. Sanchez-Muelas; dateIdentified: 2024; **Event:** samplingProtocol: Aquatic D Shaped Net; eventDate: 11/12/2024; year: 2024; month: 12; day: 11; **Record Level:** language: en; institutionID: 890.399.010-6; collectionID: RNC:77; institutionCode: Museo de entomología de la Universidad del Valle (MUSENUV); collectionCode: MUSENUV; basisOfRecord: PreservedSpecimen; **Material Entity:** preparations: whole animal (EtOH)

#### Diagnosis

**Figure 1.**
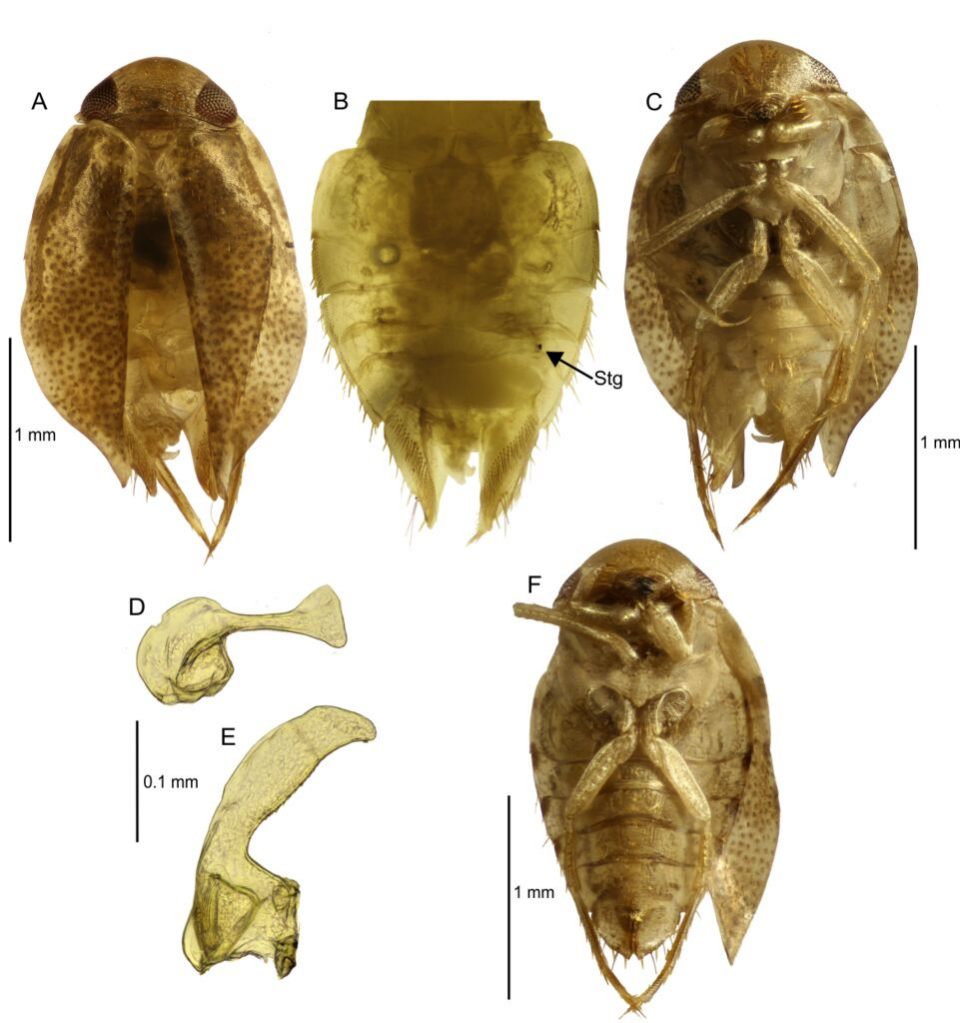
*Synaptonecta issa* (Distant, 1910). **A** Dorsal view of brachypterous male; **B** Dorsal view of the abdomen of a male and position of the strigil; **C** Ventral view of brachypterous male; **D** Left paramere; **E** Right paramere; **F** Ventral view of brachypterous female. Abbreviation: Stg: Strigil. Scales: A, C and F (1.0 mm); D and E (0.1 mm).

This species belongs to genus *Synaptonecta*, based on the antenna with three antennomeres; the scutellum not entirely covered by the pronotum; the acute hemelytral apex (Fig. 1A); the absence of a mesosternal carina; the short methaxyphus (Fig. 1C and F); and the male abdomen with dextral asymmetry, with a small strigil on the right side (Fig. 1B). *Synaptonecta issa* can be distinguished by the shapes of the male parameres, which are asymmetrical and depressed dorsoventrally (Fig. 1D and E). The right paramere (Fig. 1E) is elongated and digitiform, about 1.5 times longer than the left one. It is thicker at the basal third, followed by a constriction, then slightly widened at the apical two-thirds. The basal third has a ventral projection with longitudinal stridulatory lines at the lateral area. The mesal margin displays an almost 90° angle between the first and second thirds, while the lateral margin is convex and the apex acute. The left paramere (Fig. 1D) is small, rounded at the basal third, then strongly constricted, with the apical third clavate.

#### Distribution

**Native** (Fig. 4A): Bangladesh (Catling and Islam 2013), Cambodia (D.Polhemus 2017), India (Distant 1910, Hutchinson 1940, Wróblewski 1972, Thirumalai 2007, Saha and Gupta 2018), Indonesia (Lundblad 1933), Malaysia (Fernando 1964, Leong 1966, Fernando and Cheng 1974), Myanmar (Hutchinson 1940), Singapore (Nieser 2002), Sri Lanka (Wróblewski 1972), Thailand (Suksai et al. 2016), Vietnam (Wróblewski 1967, Ha and Tran 2021). **Exotic** (Fig. 4B): Colombia (this work), Dominican Republic (Payano-Mercado et al. 2021), New Zealand (Jansson and Meyer-Rochow 1990), United States (J.Polhemus and Rutter 1997, Epler 2006, Halbert 2015a, Halbert 2015b, Epler and Denson 2017, State of Florida Department of Environmental Protection 2019).

#### Ecology

##### Habitat

We collected the specimens in a permanent lagoon in the Pacific Region of Colombia, in a section filled with aquatic herbaceous plants, *Cabomba cf. haynessi* (Cabombaceae) and abundant in organic matter (Fig. 3). We found another micronectid, *Tenagobia signata* in the same habitat. The repeated collection of *S. issa* one year later and the presence of brachypterous, flightless specimens suggest that a population is established in the lagoon.

### Tenagobia (Incertagobia) signata

(White, 1879)

7CEE1892-8E43-5E3E-9382-FFA73AD7F249

#### Materials

- **Type status:** Other material. **Occurrence:** catalogNumber: MUSENUV33941; recordedBy: J.D. Sanchez-Muelas | J. Hernández-Benavides | R. González; individualCount: 30; sex: 9 males, 21 females; lifeStage: adult; occurrenceStatus: present; occurrenceID: C24EBEAD-538F-5FA1-B147-0846F2065E69; **Taxon:** scientificName: Tenagobia signata; kingdom: Animalia; phylum: Arthropoda; class: Insecta; order: Hemiptera; family: Micronectidae; genus: Tenagobia; specificEpithet: signata; taxonRank: species; taxonomicStatus: Species; **Location:** continent: South America; country: Colombia; countryCode: CO; stateProvince: Valle del Cauca; county: Buenaventura; locality: Citronela; verbatimLocality: Humedal. Punto 1; verbatimElevation: 27; locationRemarks: wetland near the road to Dagua River; verbatimCoordinates: 3°51'52"N 76°58'02"W; decimalLatitude: 3.86455; decimalLongitude: -76.96715; geodeticDatum: WGS84; georeferenceProtocol: GPS; **Identification:** identifiedBy: J.D. Sanchez-Muelas | J.F. Barbosa; dateIdentified: 2024; **Event:** samplingProtocol: Aquatic D Shaped Net; eventDate: 11/12/2024; year: 2024; month: 12; day: 11; **Record Level:** language: en; institutionID: 890.399.010-6; collectionID: RNC:77; institutionCode: Museo de entomología de la Universidad del Valle (MUSENUV); collectionCode: MUSENUV; basisOfRecord: PreservedSpecimen; **Material Entity:** preparations: whole animal (EtOH)

#### Diagnosis

**Figure 2.**
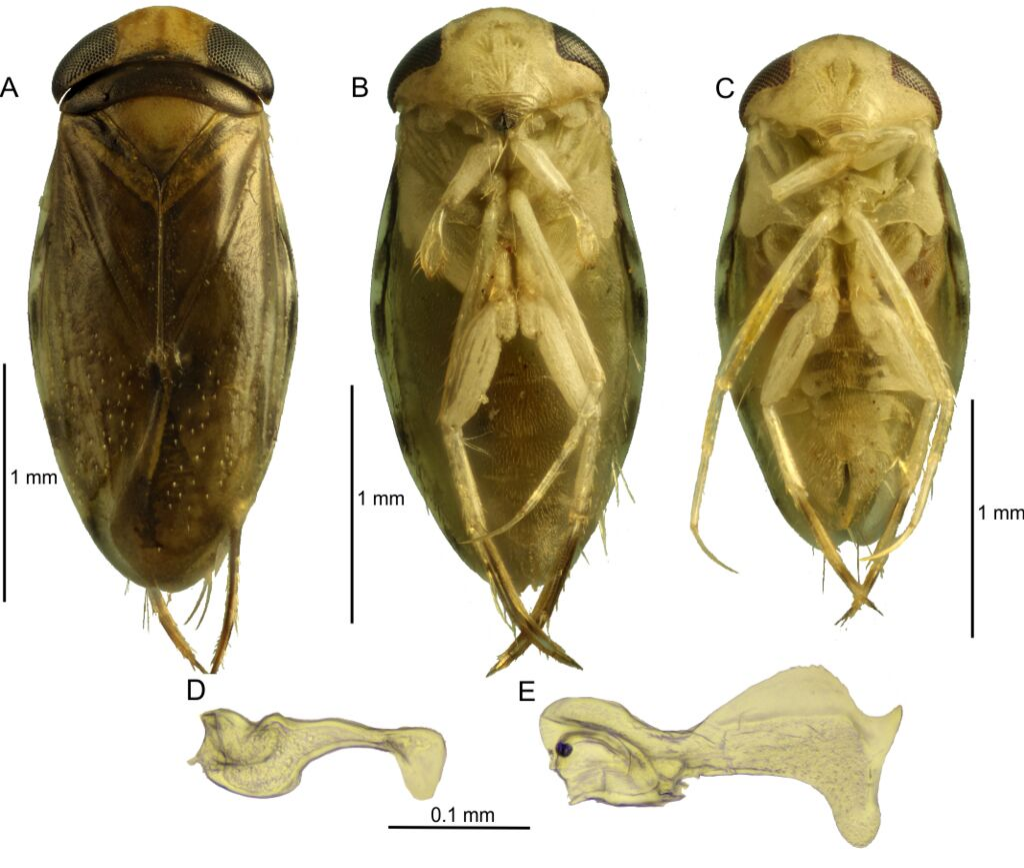
*Tenagobia signata* (White, 1879). **A** Dorsal view of macropterous female; **B** Ventral view of macropterous female; **E** Ventral view of macropterous male; **D** Left paramere; **E** Right paramere. Scales: A, B and C (1 mm); D and E (0.1 mm).

**Figure 3.**
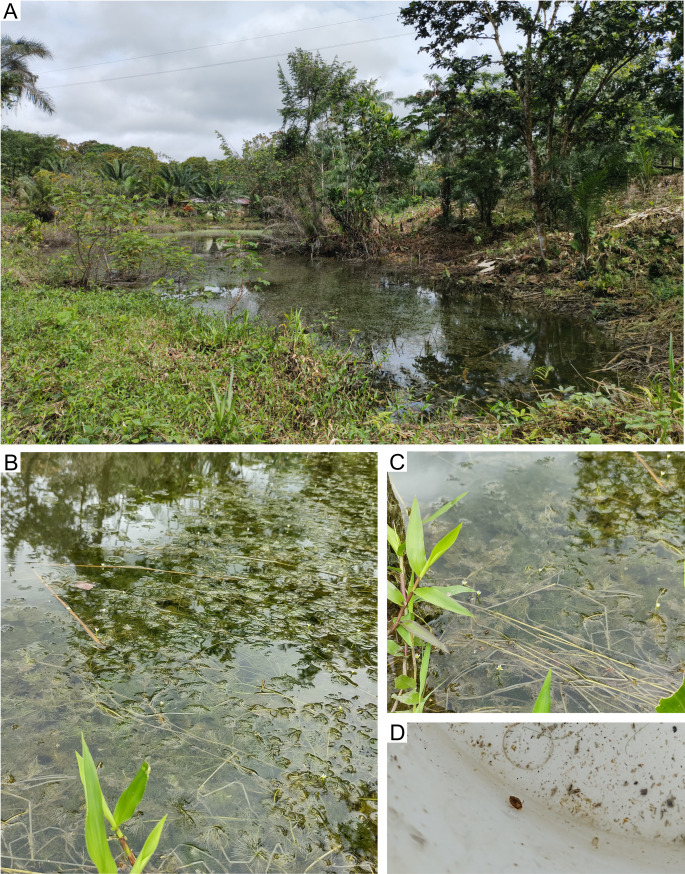
Habitat of *Synaptonecta issa* (Distant, 1910) and *Tenagobia signata* (White, 1879) in a lagoon located in Citronela, Municipality of Buenaventura, Chocoan Rainforest, Valle del Cauca Department, Colombia. **A** Panoramic view of the lagoon; **B** *Cabomba cf. haynessi* near the surface of the water; **C** Close view of *Cabomba cf. haynessi*; **D** Live *S. issa* female.

This species belongs to Tenagobia (*Incertagobia*), based on the antenna with three antennomeres; ocular index about 1.3; posterior margin of eye not sinuate, with mesal postocular space about 2.5 times the length of an eye facet; vertex suture diverging posterior to eye; pronotum not truncate at base of hemelytra; scutellum not entirely covered by the pronotum; fore femur with three or four spines on a row ventrally; mesosternal carina absent; methaxyphus short; strigil absent from right side of male abdomen; and margins of male abdominal segment VIII with four spines, three long and one short setae. *Tenagobia signata* can be distinguished from other species in the subgenus by the pala with more than 12 hair-like setae on the upper row and 15 or less on the lower row and by the shapes of the male parameres (Fig. 2D and E). The right paramere (Fig. 2E) is elongated and very sinuous, about 1.5 times longer than the left one. The basal third is enlarged, with a constricted section, followed by the straight mesal margin and the strongly dilated and barely serrated lateral margin. The apex is pickaxe-shaped and orientated dorsally. The left paramere (Fig. 2D) is less flattened than the right one, with a wider basal half and a slender bridge connecting the basal and apical sections. The apex is clavate and ventrally projected.

#### Distribution

**Figure 4.**
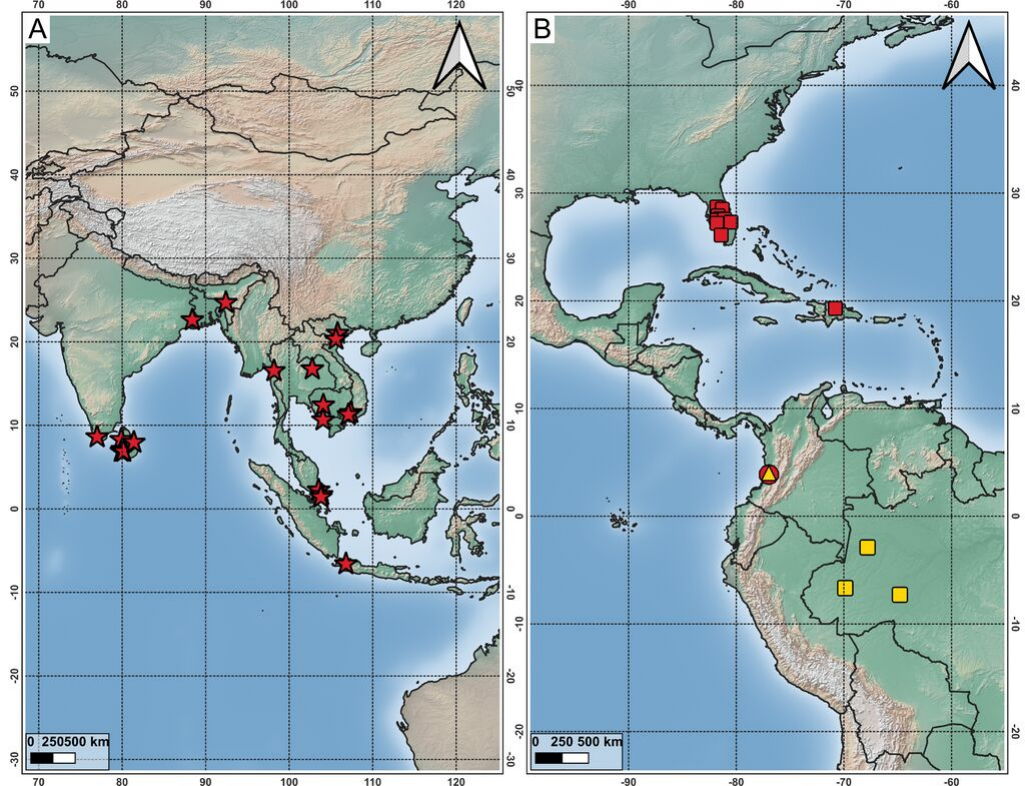
Known distribution of *Synaptonecta issa* (Distant, 1910) and *Tenagobia signata* (White, 1879). **A** Native distribution of *S. issa* in Southeast Asia; **B** Distribution of *S. issa* (introduced) and *T. signata* (native) in the Americas. Squares: Previous records of *S. issa* (red) and *T. signata* (yellow) in the Americas, Red Stars: Previous records of *S. issa* in Southeast Asia, Red Circle: New record of *S. issa*, Yellow Triangle: New record of *T. signata*.

Brazil (White 1879, Lundblad 1933, Deay 1935, Nieser 1977). Colombia (this work). See Fig. 4B.

#### Ecology

##### Habitat

This species shares the described habitat for *S. issa*.

## Identification Keys

### Key to the genera of Micronectidae recorded from the Neotropics.

**Table:** 

1	Mesosternum without a carina	Micronectinae, 2
–	Mesosternum with a distinct carina clearly surpassing apex of hind coxae	Synaptogobiinae: *Synaptogobia*
2	Antennae tri-articulate; methaxyphus short	3
–	Antennae uni-articulate; methaxyphus long	* Monogobia *
3	Pala and tibia of fore-leg fused in both sexes; male abdomen with strigil	*Synaptonecta* (Non-native)
–	Pala and tibia of fore-leg fused only in females; male abdomen without strigil	* Tenagobia *

Modified from Moreira et al. (2018) to add the genus *Synaptonecta* Lundblad, 1933.

## Discussion

Jansson and Meyer-Rochow (1990) and J.Polhemus and Rutter (1997) suggested that the introduction of micronectids into non-native areas could be related to their habit of attaching eggs to aquatic plants. These, in turn, are commonly exported to New Zealand and the United States from Singapore and other Southeast Asian countries, from where *Synaptonecta issa* is native (Ha and Tran 2021). It is plausible that the introduction pathway to South America followed a similar route, as some Asian aquatic plants are commercially available in the country; however, whether these plants are locally cultivated or only imported remains unknown. Additionally, the collection site is located less than 12 km from the port of Buenaventura, the main port in Colombia and within 2 km of the primary highway used to transport imports to major Colombian cities. In this context, the port of Buenaventura and other Colombian ports have been recognised as potential introduction pathways for alien species into the country, particularly in marine and coastal areas (Gracia et al. 2011). Maritime international trade is also suggested as the primary invasion route for the Corixid *Trichocorixa verticalis* into Mediterranean countries (Guareschi et al. 2013). However, unlike *T. verticalis*, *S. issa* does not inhabit saline waters and future molecular phylogeographic analyses could help to understand the invasion route.

We collected both *S. issa* and *T. signata* associated with the native aquatic plant *Cabomba cf. haynessi*. In Florida, J.Polhemus and Rutter (1997) found the former in a wetland dominated by *Panicum hemitomon* Schult., *Fuirena scirpoidea* Michx., *Nymphaea odorata* Aiton and *Spirogyra* sp. and in a lake abundant in *Panicum repens* L. and *Eleocharis* sp. Other records from native or non-native areas lack such details, but it is possible that *Synaptonecta* and other corixoids explore a wide range of aquatic plants for oviposition, which could facilitate their maintenance after being introduced.

The distribution and status of *S. issa* in Colombia remain unknown. However, a population of the species is likely established in Citronela, Buenaventura, as only brachypterous, flightless specimens were collected in two subsequent years, although the macropterous form of the species is also known (Hutchinson 1940). The spread of *S. issa* through the Chocó biogeographic region and Neotropics, should be investigated, as it might have happened before in Florida, where it was initially found in St. Lucie County (J.Polhemus and Rutter 1997) then moved to a wider area (Epler and Denson 2017).

## Supplementary Material

XML Treatment for Synaptonecta issa

XML Treatment for Tenagobia (Incertagobia) signata
